# Strontium ranelate as a possible disease-modifying osteoarthritis drug: a systematic review

**DOI:** 10.1590/1414-431X20187440

**Published:** 2018-06-18

**Authors:** T.A. Rodrigues, A.O. Freire, B.F. Bonfim, M.S.S. Cartágenes, J.B.S. Garcia

**Affiliations:** Centro de Ciências Biológicas e da Saúde, Universidade Federal do Maranhão, São Luís, MA, Brasil

**Keywords:** Osteoarthritis, Strontium ranelate, Treatment, Pain, Symptoms

## Abstract

Considering that osteoarthritis (OA) is the most prevalent joint disease worldwide, multiple pharmacological treatments have been proposed to alter the articular structure with potential benefit in the progression of the disease. The so-called disease-modifying OA drugs have been frequently investigated but conclusive findings are rare. Strontium ranelate (SrRan) is a drug usually prescribed to treat osteoporosis, with proven effects in decreasing the risk of fractures and possible effect in reducing the progression of OA. The objective of this review was to demonstrate the current panorama of knowledge on the use of SrRan in clinical and experimental models, clarifying its mechanisms of action and describing possible anti-nociceptive and anti-inflammatory effects. The systematic review was based on the PRISMA statement and included articles that are indexed in scientific databases. Fifteen studies were included: seven pre-clinical and eight clinical studies. Despite the limited number of studies, the results suggest a positive effect of SrRan in patients with OA, through changes in functional capacity and reduction of progression of morphological parameters and joint degradation, with moderate quality of evidence for those clinical outcomes. Novel studies are necessary to elucidate the molecular targets of SrRan, focusing on anti-inflammatory effects and histological changes promoted by SrRan, which seemed to reduce the progression of OA in the experimental and clinical studies.

## Introduction

Osteoarthritis (OA) is the most prevalent joint disease worldwide, and it directly affects the performance of daily activities. Consequently, it increases the vulnerability and functional limitations of patients, contributing to the reduction of their well-being and quality of life (1). Thus, OA is a relevant public health problem, requiring special attention (1,2).

Of the several pathophysiological phenomena that occur in OA, modification in both the structure and function of the subchondral bone begins early, implying indirect damage to the adjacent cartilage. Another important factor is that chondrocytes, osteoblasts, and osteoclasts have calcium-sensitive receptors and participate in similar physicochemical mechanisms (3).

The structure and physiology of articular cartilage as well as the inflammatory aspects of the degenerative process have been well studied (3,4). Among the pro-inflammatory mediators, tumor necrosis factor alpha (TNF-α), interleukin-1 (IL-1), and interleukin-6 (IL-6) all play essential roles in the development of pain in OA and in other inflammatory events (4). Such mediators are responsible for stimulation of prostaglandin synthesis and release of sympathomimetic amines. These cytokines have a catabolic effect, leading to the destruction of articular cartilage by inducing the release of lytic, zinc-dependent enzymes, known as metalloproteinases (collagenase, gelatinase, stromelysin). Additionally, they decrease the production of tissue inhibitory agents of metalloproteinases and plasminogen inhibitors (5). IL-1β and TNF-α inhibit the synthesis of extracellular matrix components, with IL-1β inhibiting the synthesis of aggrecan and suppressing the synthesis of collagens II and IX (constituents of cartilage), besides increasing the production of collagen I and III, resulting in poor tissue repair (6). Regulatory factors of osteoclastic activity play a significant role in the natural history of OA, especially the osteoprotegerin-RANKL (receptor activator of nuclear factor kappa-B ligand) pathway (7,8).

In this context of complex mechanisms associated with the pathophysiology of OA, the search for optimal treatment for each stage of the disease has been challenging. Most study objectives involve evaluations of non-pharmacological strategies (aimed at improving the functional state of the joint, postponing or avoiding surgical interventions) (9,10), drug therapies (such as opioid and non-opioid analgesics, anti-inflammatory drugs, chondroitin associated or not with glucosamine, diacerein, chloroquine, intra-articular hyaluronic acid, among others) (10–15), and surgical approaches for cases of clinical management failure (16).

Drugs with a probable effect on the alteration of the articular structure with potential benefit in the progression of the disease have been called disease-modifying OA drugs (DMOAD) and are frequently investigated. However, studies have not presented very conclusive findings (17).

Strontium ranelate (SrRan), an antiresorptive and bone pro-forming agent already proven effective in patients with severe osteoporosis, has been the subject of clinical and experimental studies on OA because of a probable effect on both bone turnover and inflammation associated with this disease, despite the current concern with the occurrence of cardiovascular events associated to its long-term use (18–24 [Bibr B23]). The exact mechanism of action of SrRan is not fully understood. However, regulation of bone cell differentiation, stimulation of osteoblast proliferation, and inhibition of osteoclast formation with probable apoptosis of “mature” cells, in addition to the activation of calcium-sensitive receptors have been considered as possible mediators of the pharmacological properties of this medication (8,20,25). The inhibition of osteoclastic activity by SrRan has been demonstrated to be related to the reduction in matrix metalloproteinase (MMP) synthesis and modulation of the osteoprotegerin-RANKL pathway (26).

Considering the existing evidence of strontium ranelate action on both articular cartilage and subchondral bone and the modest number of studies involving the action of this drug in OA, the objective of this review was to demonstrate the current panorama of knowledge on the subject, related to the use of SrRan in clinical and experimental models, aiming to describe possible anti-nociceptive and anti-inflammatory effects associated with the use of that drug.

## Material and Methods

The present systematic review was conducted in accordance with the guidelines of PRISMA (Preferred Reporting Items for Systematic Reviews and Meta-analyses) for checklist and construction of the flowchart in four stages (identification, selection, eligibility, and inclusion) (27). A search was carried out for articles published in national and international journals indexed in the United States National Library of Medicine (PubMed), Scientific Electronic Library Online (SciELO), Science Direct, and Biblioteca Virtual em Saúde/Centro Latino-Americano e do Caribe de Informação em Ciências da Saúde (VHL/BIREME), in September 2017. The current review was registered in International Prospective Register of Systematic Reviews - PROSPERO (CRD42017077874).

The research was based on the acronym PICOS (Patients/Intervention/Comparison/Outcomes/Study design) (28). All *in vivo* and *in vitro* models of osteoarthritis as well as participants of all ages included in clinical trials were considered eligible. In this review, we listed all available dosages of SrRan administered orally for therapeutic and prophylactic purposes in comparison with usual treatment for osteoarthritis or placebo. Regarding the outcome, we considered studies that evaluated the treatment by analysis of joint radiological alterations, besides those with histopathological analyses and inflammatory biomarkers. As for the design of the studies, original articles, both *in vivo* and *in vitro*, were considered as well as *post hoc* analyses of prospective studies. During the bibliographic research, the combination of descriptors and qualifiers, indexed in the Medical Subject Headings (MeSH) and Health Descriptors (DeCS), and certain free terms were used to construct the search strategy. The descriptors used were “arthritis” or “osteoarthritis” and “strontium ranelate” and “treatment”.

The inclusion criteria for selecting the articles were as follows: presence of the descriptors chosen in the title of the study or in the abstract, full-text articles available on the internet, publications in Portuguese or English, and studies published between January 2000 and August 2017. We excluded descriptive studies that did not provide accurate information about the method used and/or results obtained, as well as incomplete articles, reviews, editorials, comments, and studies that did not have the descriptors used in the search as the main object of the research. Articles in a language other than Portuguese or English were also excluded.

After refining the search, duplicate studies were identified and excluded. All abstracts of the remaining articles were read. In cases where reading the abstract was not enough to establish whether the article should be included considering the inclusion criteria, the article was read in its entirety.

The included studies were submitted to a critical analysis by the authors of the review through reading, focusing on the method used and the instruments for evaluating the clinical manifestations of OA, as well as the results obtained with the interventions. Details of the evaluated articles are presented as Supplementary Material, separated between experimental studies and clinical trials.

The reduced progression of the joint lesions radiologically evidenced in clinical trials was considered as a positive event related to treatment; an evaluation of the patients was conducted if the studies generated this outcome. To describe the quality of the evidence for this outcome, a GRADE (Grading of Recommendations Assessment, Development, and Evaluation) method was adopted (29). By this method, through the investigation of factors such as study limitations, inconsistency of results, inaccuracies, and publication bias, the quality of the evidence was classified into four levels: high, moderate, low, or very low.

## Results

**Figure 1.**
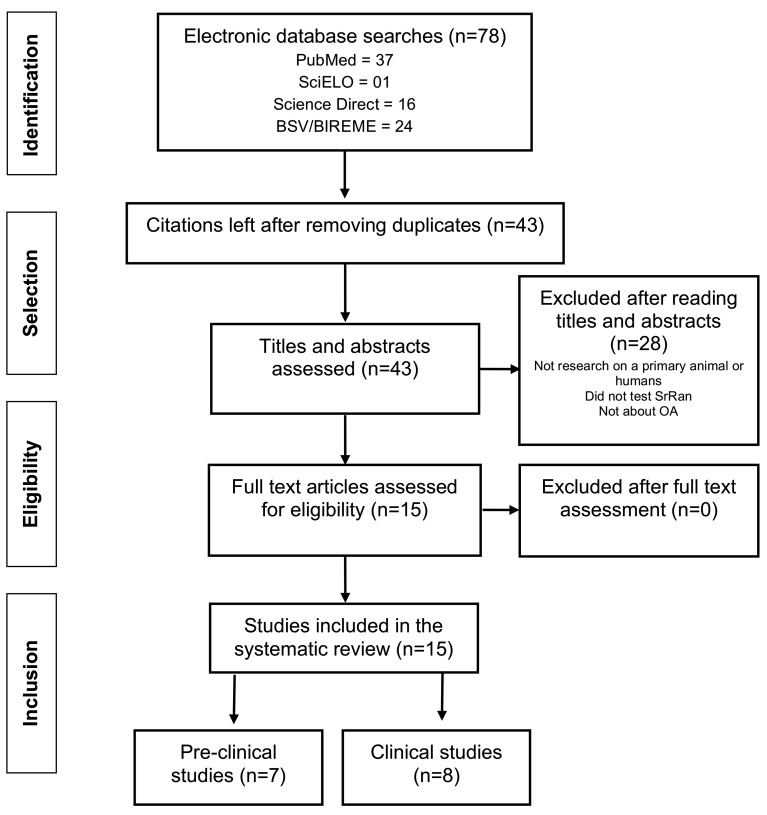
Flowchart of systemic review article search. OA: osteoarthritis; SrRan: strontium ranelate.

The search in the databases resulted in 78 articles related to the descriptors. Of these, duplicate studies were excluded, resulting in 43 studies. These studies had their abstracts read and, after a joint critical analysis by the authors, those that did not present the outcomes of interest were removed, resulting in 15 articles. These studies were read in full and divided between clinical trials and experimental studies (Figure 1). The results of the studies are summarized in Supplementary Tables S1 and S2.

The quality of the evidence of change in radiological progression with SrRan or placebo by the GRADE method is detailed in Supplementary Table S3.

## Discussion

The present systematic review sought to analyze articles related to the use of SrRan in pain management of OA, aiming to obtain the best information for its use as a disease-modifying drug.

The protective properties of strontium in bone were first described in 1959 when strontium lactate was reported as capable of decreasing pain and increasing bone density assessed radiographically in a small study of patients with osteoporosis (30). Thus, its use for the treatment of osteoporosis, for example, has been occurring for some decades (25). SrRan contains two strontium atoms, which is a bivalent calcium-like cation, in addition to an organic moiety called ranelic acid, the latter being a highly polarized molecule with no pharmacological activity (31). The atom itself has affinity for bone and, under certain conditions, a metabolism similar to that of Ca^+2^. However, despite the attraction to bone tissue, the atomic integration is still low and, theoretically, only one in ten Ca^+2^ atoms can be replaced by strontium (31).

The majority of studies on SrRan published so far involved patients with osteoporosis and promoted the developing knowledge on its probable clinical effects, which raised the hypothesis of its use in OA (18–21). Although the mechanism of action of SrRan has not yet been fully elucidated, it is possibly associated with an effect on bone metabolism, correcting the imbalance between bone resorption and bone formation observed in these osteoarticular conditions (7,8).

The preclinical studies reported in the present review have shown mixed results regarding the benefit of using SrRan in OA, especially regarding the variety of doses used and the multiple induction models employed. It is also worth noting that many positive results were obtained with administration of increased doses of SrRan (as 625 to 1800 mg·kg^−1^·day^−1^), unlikely to be transposed into clinical trials.

In a recent survey of rats that had knee OA induced by intra-articular injection of MIA (sodium monoiodoacetate), prophylactic administration of SrRan at daily doses of 25 mg/kg and post-induction use of this drug at doses of 25 and 50 mg·kg^−1^·day^−1^ did not promote improvement in mechanical hyperalgesia (assessed by the Randall Selitto test), joint incapacitation (assessed by the weight-bearing test) and motor activity (assessed by the rotarod test) (22).

Additionally, OA models with zymosan, a potent inducer of COX-2 expression, were used in an experimental study in the temporomandibular joint. Clinical evaluation by Von Frey's test showed a reduction in hypernociception with SrRan doses of 0.5, 5, and 50 mg·kg^−1^·day^−1^. Furthermore, there was a decrease in TNF-α expression with no change in leukocyte counts and IL-1β levels, suggesting an antinociceptive action by reducing that inflammatory mediator (32).

Oophorectomy has also been used for the induction of osteoporosis and osteoarthritis in rats by establishing early menopause. Either 300 or 625 mg·kg^−1^·day^−1^ of SrRan associated with vibratory stimuli, or not, were used for histological investigation of articular cartilage quality, as well as immunohistochemical analysis for caspase-3, collagen type II, TNF-α, and MMP-9 (33). It is worth mentioning that the expression of caspase-3 is related to cellular apoptosis. Metalloproteinases, in turn, are lytic enzymes responsible for extracellular matrix degradation; hence, also called matrixins. In this study, SrRan at a dose of 300 mg·kg^−1^·day^−1^ was efficient in attenuating the progression of osteoarthritis, improving the quality of the cartilaginous matrix by a direct stimulus on the synthesis of proteoglycans, preserving the cellular viability in oophorectomized rats, with reduced expression of caspase-3 and lower OARSI (Osteoarthritis Research Society International) scores. This effect was lost with daily doses of 625 mg/kg administered along with mechanical vibration. The expression of MMP-9 was not altered with the use of SrRan. Contrary to what was found in a previous study, no reduction in TNF-α expression was observed in this study (33).

Another method used in preclinical studies for induction of OA is anterior cruciate ligament transection (ACLT). One study analyzed paw elevation time (PET) and Von Frey test in groups of rats submitted to ACLT or zymosan induction, with subsequent use of SrRan at doses of 30 and 300 mg·kg^−1^·day^−1^. Additionally, cytological analysis and ELISA for TNF-α, IL-1β, and cytokine-induced neutrophil chemoattractant (CINC-1) using the synovial fluid were performed. Reduction in PET was observed in zymosan-induced models receiving SrRan, whereas in rats subjected to ACLT, there was an increase in the paw withdrawal threshold at the administered doses. It was suggested that SrRan promoted analgesia in the two OA models evaluated, associated with reduced release of cytokines TNF-α and IL-1β, but not CINC-1, at doses of 300 mg·kg^−1^·day^−1^. In the same study, reversal of analgesia promoted by SrRan with naloxone administration was observed, suggesting an opioid effect associated with the mechanism of drug action (34).

A reduction in the progression of joint structural changes was also demonstrated using SrRan in an experimental model with dogs submitted to anterior cruciate ligament transection and receiving doses of 25, 50, and 75 mg·kg^−1^·day^−1^ of the drug. Effects such as decreased depth and size of joint lesions, in addition to greater preservation of the articular collagen network were observed by histomorphometric analysis. Expression of osteochondral degradation protease genes (such as metalloproteinases and cathepsin K) and IL-1β was reduced, especially with higher doses of the drug and for longer periods of time (35).

Higher doses of SrRan (625 and 1800 mg·kg^−1^·day^−1^) were tested in mice with OA induced by meniscal injury, demonstrating an attenuation in joint degeneration. The reduction of apoptotic chondrocyte indices was proven by the TUNEL method (transferase-mediated dUTP-TMR nick end labeling assay). Using computed micro-tomography to evaluate bone mineral density, an improvement was found in the abnormality indexes in the microarchitectures of the knees investigated. Microspectroscopy determined an increase in the mineral:collagen ratio with the use of SrRan. Additionally, an increase in joint elasticity was verified through nanoindentation techniques, a dynamic test to determine the hardness of the materials. Increased expression of SOX-9 (sex-determining region Y - box 9), a transcription factor of fundamental importance in chondrogenesis, was also observed. Thus, treatment with high doses of SrRan presented positive results on the control of articular cartilage deterioration and subchondral bone remodeling (36).

Moreover, subchondral osteoblast cultures were used to investigate the action of SrRan on the bone resorption process by quantifying the expression of MMP-2, MMP-9, osteoprotegerin (OPG), and total RANKL and isoforms (26). Osteoblasts play a key role in promoting bone formation and, indirectly, modulating osteoclast differentiation through the expression of RANKL and OPG when, together with the RANK receptor, they regulate osteoclast formation and activity. RANKL is a transmembrane protein highly expressed in pre-osteoblasts, osteoblasts, periosteal cells, and osteocytes, capable of binding and activating the RANK receptor, the latter widely present in the osteoclast membrane and its precursors. After this binding, RANKL stimulates the formation, activity, and survival of osteoclasts, resulting in bone resorption. OPG, on the other hand, has high affinity for RANKL and competes for the RANK receptor on osteoclasts, preventing binding, and therefore inhibiting osteoclastogenesis (7,8). The findings of such research revealed reduced metalloproteinase expression and increased OPG synthesis in osteoblast cultures of bones with OA concentration of 1 and 2 mM SrRan, in addition to increased expression of total RANKL and isoforms. Enzymes associated with membrane RANKL cleavage, such as membrane type-1 (MT1)-MMP, ADAM17, and ADAM19 (a disintegrin and metalloproteinase domain 17 and 19), did not have their expression altered in the cultures with SrRan (26).

Although indications point to the potential benefits of SrRan in OA, its prescription for this purpose has not yet been approved by international control organizations such as the US Food and Drug Administration (FDA) and the European Medicines Agency (EMA), and the latter only allows it for the treatment of severe osteoporosis (37).

The first observations of the clinical effect of the use of SrRan in OA were derived from *post hoc* analyses of randomized trials with patients with a primary diagnosis of osteoporosis. Studies such as TROPOS (Treatment of Peripheral Osteoporosis Trial) and SOTI (Spinal Osteoporosis Therapeutic Intervention Trial) demonstrated a reduction in the radiographic progression of spinal OA in women with osteoporosis, with lower pain scores after three years of follow-up, pointing to a possible modifying effect of the SrRan on the disease. It should be noted, however, that such analyses did not demonstrate a difference in quality of life between patients who used SrRan and those who received placebo (38).

Evaluation of the effect of SrRan on subchondral bone remodeling was also performed in *post hoc* clinical trial analysis including women with osteoporosis, with or without concomitant diagnosis of OA. The levels of CTX-II (C telopeptide of type II procollagen), a urinary marker of cartilaginous degradation, and CTX-I (C telopeptide of type II procollagen), serum marker of bone resorption, were lower in SrRan users, indicating a protective action on the articular cartilaginous matrix (39).

The largest clinical research ever developed specifically in patients with OA was SEKOIA (Strontium Ranelate Efficacy in Knee Osteoarthritis Trial), a multicenter randomized, double-blind, placebo-controlled study with patients with knee OA who were treated with SrRan (40). For three years, 1683 patients of both sexes were followed-up and divided into groups that received placebo, or 1 or 2 g/day of SrRan. The primary outcome was the evaluation of radiographic changes from baseline. Secondary outcomes were the investigation of radioclinical progression, analysis of functional, pain, and urinary CTX-II scores at half-yearly intervals. Functional scores were measured by the WOMAC (Western Ontario and McMaster Universities Osteoarthritis) questionnaire, an instrument that measures different dimensions of the health status of patients with OA (with subscales for pain, stiffness, and physical function), especially in the knee and hip, and their lower indexes are associated with better algofunctional profiles (41). Pain records were made by visual analog scale. Lower radioclinical progression was observed in SrRan users, especially at doses of 2 g/day. The WOMAC and pain scores were only lower in users of 2 g/day doses of SrRan. Users of SrRan also had lower urinary CTX-II levels, confirming beneficial findings previously reported on articular cartilage turnover (40).

Several analyses were performed on subgroups of SEKOIA trial patients, giving greater weight to the evidence of the effect of SrRan on OA. An evaluation in SEKOIA patients was conducted in a subgroup that performed annual nuclear magnetic resonance, aiming to verify alterations in the global volume of cartilage of the knee and in its lateral and medial compartments (femoral and tibial components), in addition to bone marrow lesions associated to OA, demonstrating varied patterns in relation to the different regions of the joint. The daily use of 2 g of SrRan was related to a lower overall loss in articular cartilage volume, which was not observed in smaller doses in the medial component of the knee. In the lateral compartment, the loss of cartilage was reduced in the first and second years of patients receiving 2 g/day and from the second year in patients with doses of 1 g/day. Both doses were shown to be effective in decreasing bone marrow lesions related to OA (42).

Additional interpretations with radiography were also performed aiming at the identification of responders to SrRan treatment from the SEKOIA trial, based on the reduction of joint narrowing progression, with three cut-off levels (joint reduction ≥-0.1, -0.2 or -0.3 mm). Preservation of articular cartilage was observed in comparison with placebo, with NNT=13 (number needed to treat) with use of 1 g/day and NNT=9 with 2 g/day to promote joint space reductions ≥ -0.3 mm (43).

Another subgroup of SEKOIA trial patients submitted to hand radiography to assess OA in this joint component showed a slight radiological progression for the placebo, with no statistical difference in the use of 1 or 2 g/day. There was a trend toward lower pain scores with 2 g/day, especially in more severe cases of hand OA, determined through FIHOA (Functional Index for Hand Osteoarthritis) and AUSCAN (Australian-Canadian questionnaire) clinical classification, with the latter evaluating pain patterns, joint stiffness, and physical function (44).

In a study of response analysis for the demonstration of clinical effect magnitude of SrRan in SEKOIA trial patients WOMAC, OMERACT-OARSI (Outcome Measurements in Rheumatology - Osteoarthritis Research Society International) scales and MPCI (Minimal Perceptible Clinical Improvement) and MCII (Minimal Clinical Important Improvement) criteria were used for response analysis (45). OMERACT-OARSI criteria are internationally validated for response analysis of clinical trials in OA, evidencing effects on symptoms through dichotomous responses to specific questions (46,47). Other indexes used in analyses of response to the WOMAC score are MPCI and MCII, which determine, respectively, the lowest values at which the patient begins to perceive clinical improvement and from which the patient classifies this improvement as important. These values had their thresholds previously determined (48,49). In this study, no effect on symptoms was observed for daily doses of 1 g of SrRan over placebo. Doses of 2 g/day led to better WOMAC scores for pain, in addition to a response above the MPCI threshold in the overall WOMAC score (for pain, stiffness, and physical function) and above the MCII threshold in the WOMAC score for physical function (45).

In SEKOIA patients, in whom meniscal extrusion and/or bone marrow lesion were identified in the medial knee compartment, there was a greater reduction of joint space and loss of cartilage when using placebo, in contrast to the use of 2 g/day of SrRan, which reduced the progression of OA, with less loss of cartilage in the medial plateaus. Such findings are relevant because they reinforce SrRan's protective effect of articular cartilage, even in cases of greater severity, with meniscal lesions and already established subchondral bone remodeling (50).

The most common side effects with SrRan are nausea and diarrhea, which usually appear at the beginning of treatment and disappear after approximately three months of use. The drug may also be rarely related to certain serious and potentially lethal physiological changes, such as the skin reactions Stevens-Johnson syndrome and toxic epidermal necrosis (51). It has been observed that the risks of using SrRan appear to be similar to the benefits, whereas the most serious adverse events reported were increased risk of venous thromboembolism, pulmonary embolism, and myocardial infarction (24). Therefore, caution is recommended in the prescription of the drug to patients with uncontrolled hypertension, history of ischemic heart disease, peripheral arterial disease, and cerebrovascular disease. In such situations, the use of bisphosphonates such as alendronate, risendronate, and zolendronate (24,52,53) are better options. In terms of relevance, as calcium plays a key role in the electrophysiology of the cardiac muscle and electrocardiographic abnormalities are known consequences of the plasma variations of this element, strontium has a potential arrhythmogenic effect (54). However, doses of 4 g/day have been shown to be safe, without electrocardiographic repercussion after use for 15 days. In addition, no change in the QT interval has been reported for the population using 2 g/day dose (54).

In contrast, a study carried out in the United Kingdom found no evidence for an increased risk of myocardial infarction with the use of SrRan in women diagnosed with osteoporosis compared to the non-use of this drug (55). In a cohort study, SrRan also was not associated with an increased risk of acute coronary syndrome or any other cause of mortality (56).

SrRan is not approved by the FDA for use in the US, but the EMA has endorsed its use for a long time. However, there is a recent recommendation for discontinuation of the drug marketing in Europe by the manufacturer, considering the adverse effects described above (52,57). The Brazilian National Agency of Sanitary Surveillance (ANVISA) and other regulatory agencies in Latin America, in turn, maintain SrRan registry for treatment of severe osteoporosis in men and women, especially in cases in which other anti-osteoporosis medications are inappropriate (58). Although there are still issues associated with adverse effects related to drug use, recent publications, still seeking a better understanding of the mechanisms involved in the action of SrRan, continue to aggregate important information about its clinical effects, as described in the present review (22,26,32–36).

Despite the limited number of studies available, the results described in this review suggest a positive effect of the use of SrRan in patients with OA, through changes in functional capacity and reduction of progression of morphological parameters and joint degradation. Moderate quality of evidence for this outcome was observed, possibly due to diversity of OA phenotypes, in addition to the differences among the patients included in the analysis of this endpoint. This property attributed to SrRan is compatible with its pharmacological effect obtained through experimental studies: improvement of the quality of the cartilaginous matrix and viability of the chondrocytes, as well as endpoints involving hypernociception and joint discomfort. However, more evidence is required, especially since most of the findings relate to one or a few randomized clinical trials. It is necessary to reinforce the signs of articular action of SrRan through novel studies to elucidate the molecular targets of this drug, focusing on anti-inflammatory effects and histological changes promoted by SrRan, which seemed to reduce the progression of OA in the experimental and clinical studies.

## Supplementary Material

Click here to view [pdf].
